# Antimicrobial Activity of NCR Plant Peptides Strongly Depends on the Test Assays

**DOI:** 10.3389/fmicb.2018.02600

**Published:** 2018-10-30

**Authors:** Attila Farkas, Bernadett Pap, Éva Kondorosi, Gergely Maróti

**Affiliations:** Institute of Plant Biology, Biological Research Center of the Hungarian Academy of Sciences, Szeged, Hungary

**Keywords:** nodule-specific cysteine-rich plant peptides (NCR peptides), antibiotics, antimicrobial agents, colorimetric resazurin microdilution assay, drop plate method

## Abstract

The symbiosis specific NCR247 and NCR335 cationic plant peptides of *Medicago truncatula* have been shown to exert antimicrobial activity against a wide range of microbes. However, their antimicrobial efficiency is clearly limited by divalent cations. Here, the antibacterial and antifungal activities of NCR247 and NCR335 peptides were compared to those of the well-characterized peptide antibiotics polymyxin B and the aminoglycoside streptomycin on three model microbes, *Escherichia coli*, *Bacillus subtilis* and *Saccharomyces cerevisiae* as representatives of Gram-negative and Gram-positive bacteria as well as eukaryotic fungi. The aim of the study was to assess how the killing efficiency of these peptides depends on various, widely used antimicrobial susceptibility assays. Validated resazurin microdilution assay was used to determine minimal growth inhibitory concentrations in three general test media (MHB, MHBII and low-salt medium LSM). Bactericidal/fungicidal activities were determined by the commonly used drop plate assay. The natural plant peptides showed distinct characteristics, NCR247 had a generally high sensitivity for Ca^2+^ and Mg^2+^ in the medium, while NCR335 proved to be a robust and strong antimicrobial agent with comparable efficiency values to polymyxin B. Activity data were confirmed visually, both NCR247 and NCR335 treatments at minimal bactericidal concentrations induced complete disruption of the membranes and provoked cell lysis on all tested microorganisms as observed by scanning electron microscopy.

## Introduction

Bacterial and fungal pathogens (the ethological agents of human infections) represent one of the major threats for human health. The widespread overuse and misuse of antibiotics cause serious environmental problems and the evolvement and fast spread of antibiotic resistant microbes (Jorgensen and Ferraro, 2009) result in raising numbers of mortality every year (Nathan and Cars, 2014). Most of the current antimicrobials have natural origin deriving from microbes, plants or animals (Bérdy, 2005).

The innate immune system of eukaryotes protects the host from microbial infections (Aderem and Ulevitch, 2000). Either in plants and animals microbe-associated pathogen-specific molecules such as lipopolysaccharides (LPS) are recognized by pattern recognition receptors (PRRs) (Reddick and Alto, 2014). This interaction results in the activation of multiple signaling processes including the production of effector molecules called host defense peptides (HDPs) and/or antimicrobial peptides (AMPs) (Steinstraesser et al., 2009). Plants have an exceptionally broad range of defense mechanisms to counter abiotic and biotic stresses (biological and chemical effects) such as heavy metals, pollutants and microbial pathogens (Tam et al., 2015). The plant innate immune system responds to infections with specific patterns of pathogenesis-related gene expression, various classes of AMPs including defensins, thionins and transfer proteins are expressed (Sels et al., 2008).

Antimicrobial peptides’s are categorized based on their secondary structure, which is amphipathic in design due to spatially organized clusters of hydrophobicity and cationic character. AMPs are highly diverse 10–75 amino acid long peptides, positively charged between+2 and+9 contain abundant positively charged amino acids (lysine and arginine) and comprised of > 30% hydrophobic residues (Maróti et al., 2011; Farkas et al., 2017). The positive charge of AMPs together with their amphipathic structure enable them to interact with the negatively charged microbial membranes (Peters et al., 2010; Bahar and Ren, 2013). Antimicrobial peptides can be classified according to their specific mode of actions interfering with cell wall synthesis, protein, DNA or RNA synthesis or inhibiting various metabolic pathways or cell cycle (Hancock and Sahl, 2006; Hale and Hancock, 2007; Hilpert et al., 2010; Maróti and Kondorosi, 2014).

Plants maintain various types of relationships with microorganisms. A crucial aspect of the plant defense systems is the ability to distinguish between pathogens and beneficial interacting microorganisms (Maróti et al., 2015). The short cysteine-rich antimicrobial peptides called defensins and defensin-like peptides play crucial role in fine-tuning the interactions with both the harmful and the commensal/symbiotic bacterial populations. Most plant defensins are secreted, typically 45 to 70 amino acid long peptides stabilized by four conserved disulphide bonds. A large number of defensins were identified in many different plants, however, only a few of these immune peptides were characterized in detail. Defensins possess antimicrobial - primarily antifungal – character, and were also shown to modulate microbial growth to the benefit of the host plants (Thomma et al., 2002; Lay and Anderson, 2005; Aerts et al., 2008).

Leguminous plants such as *Medicago truncatula* are able to establish symbiotic interactions with soil rhizobium bacteria and form a new plant organ, the nitrogen-fixing root nodule. Inside the host plant cells the bacteria are terminally differentiated to polyploid, non-dividing, non-cultivable nitrogen-fixing bacteroids (Mergaert et al., 2006; Kondorosi et al., 2013). In the inverted repeat-lacking clade (IRLC) legumes irreversible differentiation of the endosymbionts is controlled by symbiosis-specific plant peptides (Mergaert et al., 2003; Alunni et al., 2007). In the *M. truncatula* plant close to 700 genes code for secreted nodule-specific cysteine rich peptides (NCRs) that are targeted to the endosymbionts and interact either with the bacterial membranes or enter to the cytosol with multiple bacterial targets. The *NCR* genes are exclusively expressed in the rhizobium-infected nodule cells (Roux et al., 2014). The mature NCR peptides are 20–50 amino acid long which exhibit high divergence in their amino acid composition and sequence except for the presence of four or six cysteine residues at conserved positions. Based on the highly diverse primary sequences, NCRs can be either cationic, neutral, or anionic peptides, cationic NCRs resemble defensins (Mergaert et al., 2003; Farkas et al., 2014; Maróti et al., 2015). In previous reports it was shown that synthetic cationic NCR247 and NCR335 peptides had strong antimicrobial effect on a variety of bacteria and fungi, and their killing effect was realized through membrane disruption and combined intracellular actions (including protein synthesis) (Tiricz et al., 2013; Ördögh et al., 2014; Farkas et al., 2017).

Reproducible standard monitoring of antimicrobial efficiency is a crucial first step toward the development of novel antimicrobial agents. A variety of validated and unique research wet-lab approaches are used to determine the *in vitro* antimicrobial activity of antimicrobial drug candidates. The most established methods include the measurement of inhibition zones in disk-diffusion test, broth turbidity-based measurements and the counting of colony-forming units (after serial dilutions) (Jorgensen and Ferraro, 2009). There are recommendations for determination of the minimal inhibitory concentration (MIC) and minimum bactericidal concentration (MBC) by the Clinical and Laboratory Standards Institute (CLSI) or by The European Committee on Antimicrobial Susceptibility Testing (EUCAST) (Arikan, 2007; CLSI, 2015). The procedure involves the preparation of twofold dilutions of the antimicrobial agent in a liquid growth medium dispensed in tubes containing a minimum volume of 2 mL (macrodilution) or smaller volumes using 96-well microtiter plate (microdilution). The MIC is the lowest concentration of the antimicrobial agent that completely inhibits growth of the microorganism in tubes or microdilution wells as detected by unaided eye or by the Alamar blue (resazurin) colorimetric growth indicator assay (Castilho et al., 2015). The determination of minimum bactericidal concentration (MBC) or minimum fungicidal concentration (MFC), also known as the minimum lethal concentration (MLC), is the most common estimation of bactericidal or fungicidal activity. The MBC is defined as the lowest concentration of antimicrobial agent required to kill 99.9% of the final/control inoculum after incubation for 24 h (Clinical and Laboratory Standards Institute, 1999). Non-standard methods such as the drop plate method (DP) and the spread plate method (SP) are also suitable to determine the microbial elimination efficiency of an antimicrobial drug (Chen et al., 2003). The drop plate method exhibits a number of advantages over the spread plate (SP) method. The plating and counting in DP require less labor time and relatively few supplies. A bibliographic-database search shows that the drop plate method has been widely used, although it is not considered as standard approach (Herigstad et al., 2001). To further study the antimicrobial effect in depth, time-based screening methods are recommended, which provide additional information on the nature of the inhibitory effect such as bactericidal or bacteriostatic effects. On-line time-resolved kinetic analyses allow to monitor microorganisms growth in time- and concentration-dependent manner (Theophel et al., 2014). In addition, microscopic analyses can provide valuable information on the mechanism of antimicrobial activities (Farkas et al., 2017).

Our aim was to assess the antimicrobial effect of the symbiotic NCR247 and NCR335 plant peptides together with two classical antibiotics, streptomycin (STM) and polymyxin B, a cyclic antimicrobial peptide (PMB) with different methods on *Escherichia coli*, *Bacillus subtilis* and the budding yeast *Saccharomyces cerevisiae* as representative model microbes. Activity results were supported by scanning electron microscopy observation.

## Materials and Methods

### Microbial Strains and NCR Peptides

*Escherichia coli* ATCC 8739, *B. subtilis* ATCC 11774 and *S. cerevisiae* ATCC 9763 were purchased from DSMZ culture collection. NCR247 and NCR335 were synthesized by conventional solid phase peptide synthesis at> 95% purity, synthesis was done by ProteoGenix SAS (France), vendor provided data of peptide characterization including HPLC and Mass Spectrometry data. The molecular weights were confirmed by mass spectrometry. The lyophilized peptides were dissolved in phosphate-buffer (pH = 7.4) to a concentration of 10 mg/ml and stored at −20°C.

### Growth Media

The bacteria and yeast cultures were maintained in cryo-tubes at −80°C. Working inocula of bacteria were prepared on Luria-Bertani (LB) agar plates at 37°C while *S. cerevisiae* on Yeast Extract-Peptone-Dextrose (YPD) agar plate at 30°C (Lennox, 1955; Sherman, 1991). Mueller Hinton Broth (MHB) (Difco), Mueller Hinton II Broth (MHBII) (Difco) and a modified low-salt medium (LSM) composed of 5 mM K_2_HPO_4_, 100 μM MgSO_4_, 10 μM FeCl, 0.2 μM CoCl_2_, 0.2 μM CuSO_4_, 4 μM Na_2_MoO_4_, 1 μM H_3_BO_3_, 0.2 μM KI, 1 μM ZnSO_4_, 0.2 μM MnSO_4_, 2% glucose, 7.5 mM (NH_4_)_2_SO_4_, 0.2% asparagine, 100 μg/mL yeast extract, 40 μg/mL methionine, 4 μg/mL myo-inositol, 0.4 μg/mL biotin, 2 μg/mL thiamine-HCl and 0.4 μg/mL pyridoxine-HCl were used for the broth resazurin microdilution assay (Spelbrink et al., 2004; Ördögh et al., 2014).

### Determination of the Minimal Inhibitory Concentration (MIC) and Minimal Bactericidal Concentration (MBC) Using Broth Resazurin Microdilution Assay

Single bacterial colonies from the bacterial strains were grown in MHB, MHBII and LSM media at 37°C overnight while single yeast colonies in MHB, MHBII with 2% glucose and LSM medium at 30°C overnight. These starter cultures were diluted and grown in the same medium until OD_600_ = 0.5 – 1.0. The number of colony-forming units was determined, and the bacterial cultures were adjusted to 5 × 10^6^ cfu/mL while the yeast cultures to 1 × 10^5^ cfu/mL. Plates were prepared under aseptic conditions. Using sterile 96-well plates 90 microliters (μL) of sterile MHB, MHBII or LSM were added into each well of the first row serving as negative controls. 50 μL sterile MHB, MHBII, or LSM were added to all other wells. Dilution series of NCR247, NCR335, polymyxin B (PMB) and streptomycin (STM) were prepared (0.1, 0.5, 1, 2, 4, 8, 16, 32, 64, and 128 μM) and added to the wells. 10 μL of resazurin indicator solution (0.1 % diluted in appropriate medium) was added into each well. Finally, 10 μL of bacterial and yeast suspensions (5 × 10^6^ and 1 × 10^5^ cfu/mL, respectively) were added to each well to achieve a concentration of 5 × 10^5^ cfu/mL final bacterial and 1 × 10^4^ cfu/mL final yeast inocula. The plates were prepared in triplicate and incubated for 20 h. The color change from purple to pink or colorless (indicating normal growth) were recorded by visual observation. The lowest peptide/PMB/STM concentration showing growth inhibition (influencing color change) was considered as the MIC value (Wiegand et al., 2008; Balouiri et al., 2016). For the determination of MBC 100 μL cultures from the wells exhibiting no growth were plated. The lowest concentration where no bacterial growth was detected was considered as MBC (Kang et al., 2011). Three independent experiments were performed, each with three biological replicates.

### Growth Curve Analysis

Cell cultivations and sample preparations were performed in sterile 96-well standard microtiter plates (Sarstedt). 100 (μL) of sterile MHB, MHBII or LSM were added into each well of the first row serving as negative controls. 90 μL of sterile MHB, MHBII or LSM and 10 μL of bacterial and yeast suspensions (5 × 10^6^ and 1 × 10^5^ cfu/mL, respectively) were added into each well of the second row serving as positive controls. Dilution series of NCR247, NCR335, polymyxin B (PMB) and streptomycin (STM) were prepared (0.1, 0.5, 1, 2, 4, 8, 16, 32, 64, and 128 μM) and added to the wells. Finally, 10 μL of bacterial and yeast suspensions (5 × 10^6^ and 1 × 10^5^ cfu/mL, respectively) were added to each well to achieve a final bacterial concentration of 5 × 10^5^ cfu/mL and yeast concentration of 1 × 10^4^ cfu/mL. Cell cultivation and growth analysis were performed using a HIDEX Sense multimode microplate reader equipped with a high sensitivity CCD spectrograph. Bacteria in microplates were incubated at 37°C while yeast at 30°C with normal orbital shaking. Measurements were performed in 145 cycles with 10 min intervals resulting in a total of 24 h duration. The background readings were also measured as blank at each sampling time, all readings were normalized. Three parallel measurements were performed for each sample.

### Determination of the Complete Elimination Concentrations (CE values) Using Drop Plate Method

Bacterial strains were grown in 10 mL of liquid Luria Broth (LB) at 37°C overnight and *S. cerevisiae* were grown in 10 mL of liquid Yeast Extract-Peptone-Dextrose (YPD) at 30°C. These starter cultures were diluted in 10 mL LB and YPD to OD_600_ = 0.05 and grown until mid-logarithmic phase (OD_600_ = 0.5 – 0.8). The optical density was set to OD_600_ = 0.1 in 20 mM potassium phosphate buffer, pH 7.2 (PB) for the antimicrobial tests. For *E. coli*, the OD_600_ of 0.1 represents 6.4 × 10^7^, for *B. subtilis* 4.2 × 10^6^ and for *S. cerevisiae* 3.6 × 10^5^ cfu/mL. Dilutions were made to obtain bacterial cultures of 5 × 10^5^ cfu/mL and yeast culture of 1 × 10^4^ cfu/mL. The antimicrobial activities were determined according to the guidelines of the drop plate method (Herigstad et al., 2001; Chen et al., 2003). Peptides were serially diluted in PB buffer. 50 μL of bacterial and yeast cultures were mixed with 50 μL NCR peptide / STM / PMB at 0.1, 0.5, 1, 2, 4, 8, 16, 32, 64, and 128 μM final concentrations and incubated at room temperature for 2 h (without shaking) and then 10 μL were dropped on LB and YPD agar plates. The drops were absorbed to agar and the plates were incubated at inverted position overnight (Farkas et al., 2017). Three independent experiments were performed, each with three biological replicates.

### Scanning Electron Microscopy (SEM)

Mid-logarithmic phase bacteria (5 × 10^5^ cfu/mL) and yeast (1 × 10^4^ cfu/mL) were incubated in LSM with MIC, MBC of NCR peptides / STM / PMB for 20 h. Cells were then fixed with 2.5% (v/v) glutaraldehyde and 0.05 M cacodylate buffer pH 7.2 in phosphate buffered saline (PBS pH 7.4), and post-fixed with 0.1% osmium tetroxide in PBS for 1 h. 5 μL of the above bacterial and yeast suspensions were spotted on a silicon disk coated with 0.01% Poly-L-Lysine. The filters were washed twice with PBS and dehydrated with a graded ethanol series (30%, 50%, 70%, 80%, and 100% ethanol, each for 15 min). Untreated bacterial and yeast cells were processed in the same way and served as controls. The samples were dried with a critical point dryer, followed by 12 nm gold coating and observed under a JEOL JSM-7100F/LV scanning electron microscope).

## Results

### Determination of Minimal Inhibitory Concentration (MIC) and Minimal Bactericidal Concentration (MBC) Using Colorimetric Resazurin Microtiter Plate Based Antimicrobial Assay

Antimicrobial activity of NCR247 and NCR335 peptides against model strains of *E. coli, B. subtilis* and *S. cerevisiae* has been investigated and compared to that of two well-characterized antimicrobials, peptide antibiotics polymyxin B (PMB) and aminoglycoside streptomycin (STM). In the standard resazurin-based colorimetric assay, all sterility control wells for all tested microorganisms remained blue after 24 h incubation. In contrast, all growth control wells (containing growth medium and microorganisms) for all tested microorganisms changed from blue to pink or to colorless indicating normal growth. For *E. coli* the MIC values of PMB were 0.5 μM in all tested media, while the MBC values were 1 μM in LSM and MHB media and 0.5 μM in MHBII. STM inhibited cell growth most effectively in LSM at 1 μM and eliminated all *E. coli* bacteria at a concentration of 2 μM (MBC) in this medium. The MIC values of STM were 16 μM in MHB and 8 μM in MHBII, while the MBC was fourfold of its MIC in MHB and MHBII. The MIC values of NCR247 and NCR335 were 16 μM in LSM broth against *E. coli*. The MBC of NCR247 in LSM was 32 μM and remained 16 μM (equal to MIC) for NCR335. In MHB and MHBII medium, the MIC value for NCR247 was 128 μM. NCR335 had a stronger antimicrobial effect on *E. coli* described with a MIC of 64 μM. NCR247 and NCR335 had highly similar MBC values of 128 μM (Table 1).

**Table 1:** Minimal inhibitory concentration (MIC) values determined by colorimetric assay (resazurin) and minimal bactericidal concentrations (MBC) determined by conventional plating method on *E. coli*.

*Escherichia coli*								
Peptide/Antibiotics	PMB		STM		NCR247		NCR335	
	MIC	MBC	MIC	MBC	MIC	MBC	MIC	MBC
MHB	0.5 μM	1 μM	16 μM	64 μM	128 μM	128 μM	64 μM	128 μM
MHBII	0.5 μM	0.5 μM	8 μM	32 μM	128 μM	128 μM	64 μM	128 μM
LSM	0.5 μM	1 μM	1 μM	2 μM	16 μM	32 μM	16 μM	16 μM

*MIC and MBC values were determined for the NCR247 and NCR335 peptides and for PMB and STM using a standard, validated method in MHB, MHBII and LSM media.*

In the case of *B. subtilis* the MIC values of PMB were 1 μM in all tested media while MBC was 64 μM in MHB and LSM, and 16 μM in MHBII. STM in MHB did not inhibit the growth of *B. subtilis* up to 128 μM but in MHBII broth its MIC was 8 μM and its MBC 128 μM. In LSM the MIC of STM was 2 μM and the MBC was 128 μM. The MIC values of NCR247 in MHB and MHBII media were 16 μM, its MBC values in the same media were 64 μM. In LSM medium NCR247 inhibited cell growth at 8 μM (MIC) and killed all *B. subtilis* at 16 μM (MBC). The MIC of NCR335 was 32 μM in MHB and no killing effect was observed up to 128 μM. In MHBII and LSM media the MIC and MBC of NCR335 were 8 μM (Table 2).

**Table 2:** Minimal inhibitory concentration (MIC) values determined by colorimetric assay (resazurin) and minimal bactericidal concentrations (MBC) determined by conventional plating method on *B. subtilis*.

*Bacillus subtilis*								
Peptide/Antibiotics	PMB		STM		NCR247		NCR335	
	MIC	MBC	MIC	MBC	MIC	MBC	MIC	MBC
MHB	1 μM	64 μM	> 128 μM	> 128 μM	16 μM	64 μM	32 μM	> 128 μM
MHBII	1 μM	16 μM	8 μM	128 μM	16 μM	64 μM	8 μM	8 μM
LSM	1 μM	64 μM	2 μM	128 μM	8 μM	16 μM	8 μM	8 μM

*MIC and MBC values were determined for the two NCR peptides and for PMB and STM using a standard, validated method in MHB, MHBII and LSM media.*

The MIC and MBC values of PMB were 32 μM for *S. cerevisiae* in both MHB media. PMB inhibited cell growth at 4 μM and killed all *S. cerevisiae* at 8 μM in LSM medium. As expected, STM was not able to inhibit yeast growth up to 128 μM in any of the tested media. Similarly, NCR247 and NCR335 had no effect on the growth of *S. cerevisiae* in MHB and MHBII broth. However, NCR247 inhibited yeast growth at 8 μM (MIC) and killed all cells at 16 μM (MBC) in LSM medium. The growth inhibition was 8 μM (MIC) of NCR335 in LSM medium, while NCR335 MBC was detected at a much higher concentration of 64 μM (Table 3).

**Table 3:** Minimal inhibitory concentration (MIC) values determined by colorimetric assay (resazurin) and minimal bactericidal concentrations (MBC) determined by conventional plating method on *S. cerevisiae*.

*Saccharomyces cerevisiae*								
Peptide/Antibiotics	PMB		STM		NCR247		NCR335	
	MIC	MBC	MIC	MBC	MIC	MBC	MIC	MBC
MHB	32 μM	32 μM	> 128 μM	> 128 μM	> 128 μM	> 128 μM	> 128 μM	> 128 μM
MHBII	64 μM	64 μM	> 128 μM	> 128 μM	> 128 μM	> 128 μM	> 128 μM	> 128 μM
LSM	4 μM	8 μM	> 128 μM	> 128 μM	8 μM	16 μM	8 μM	64 μM

### Analysis of Microbial Growth Dynamics in Susceptibility Tests

The effects of PMB, STM, NCR247 and NCR335 on the growth dynamics of the applied model strains of *E. coli, B. subtilis* and *S. cerevisiae* were continuously monitored using microtiter plate assay. The results corroborated well with the standard MIC measurement data with minor variations (Supplementary Figures 1–9). The growth curve analysis revealed that the 16 μM MIC value determined for STM against *E. coli* in MHB was correct; however, after 20 h (the endpoint of the standard MIC assay) *E. coli* started propagating in this medium resulting in a modified MIC value of 32 μM (Supplementary Figure 1B). NCR335 was shown to inhibit *E. coli* growth already at 32 μM in the first 15 h of the assay, then the cells showed normal growth supporting the originally detected MIC value of 64 μM both in MHB and MHBII (Supplementary Figures 1D, 2D). Another interesting phenomenon revealed exclusively by the growth curve analysis was the concentration-dependent delay of *E. coli* growth exhibited by STM in MHBII (Supplementary Figure 2B). NCR247 and NCR335 showed similar concentration dependent effects on *E. coli* in LSM. The endpoint MIC assay indicated 16 μM MIC for both plant peptides on *E. coli*, however, the growth curves revealed partial inhibition exerted by both peptides already at 8 μM, 4 μM and even at 2 μM in this medium (Supplementary Figures 3C, D). All MIC values were confirmed for *B. subtilis* by the growth analyses. Nonetheless, the kinetic measurements shed light on some interesting characteristics, i.e., the plant peptides caused delayed start of Bacillus propagation in MHB already below their MIC (8 μM for NCR247 and 16 μM for NCR335) (Supplementary Figures 4C, D). The interesting susceptibility features of *B. subtilis* for STM was highlighted by the growth curve analysis. STM caused disturbed growth in MHBII and especially in LSM even below the low MIC values, while resistance of *B. subtilis* to STM in MHB was also confirmed (Supplementary Figures 4B, 5B, 6B). MIC data measured for *S. cerevisiae* were supported by the growth analyses. NCR335 was again shown to exert a concentration-dependent growth delay when applied in MHB and MHBII (Supplementary Figures 7D, 8D).

### Determination of Complete Elimination Values (CE) Using Drop Plate Technique

Using the drop plate approach PMB and NCR335 fully eliminated colony formation of *E. coli* at 0.5 μM and 1 μM, respectively (Table 4). The complete elimination of *E. coli* required 16 μM NCR247 and the CE value of STM against *E. coli* was 32 μM. *B. subtilis* was highly tolerant to STM and PMB using this approach (both CE values were 128 μM), while this Gram + bacterium showed clear sensitivity to the plant peptides; NCR335 eliminated all *B. subtilis* at 2 μM while NCR247 had a CE value of 16 μM for this bacterium. Again, *S. cerevisiae* was fully resistant to STM, while PMB and NCR335 peptides completely eliminated fungi cells at 0.5 μM and 1 μM, respectively. NCR247 treatment resulted in complete elimination (CE) of yeast at 32 μM (Table 4).

**Table 4:** Complete elimination concentrations (CE) on *E. coli, B. subtilis* and *S. cerevisiae* using drop plate method.

Drop plate method			
Peptide / Antibiotics	Concentration of complete elimination (CE)		
	Gram-negative	Gram-positive	Fungi
	*Escherichia coli*	*Bacillus subtilis*	*Saccharomyces cerevisiae*
NCR247	16 μM	16 μM	32 μM
NCR335	1 μM	2 μM	1 μM
Polymyxin B (PMB)	0.5 μM	128 μM	0.5 μM
Streptomycin (STM)	32 μM	128 μM	NE

*NE: no effect up to 128 μM.*

### Scanning Electron Microscopy Confirmed the Antimicrobial Activity Data

**FIGURE 1:**
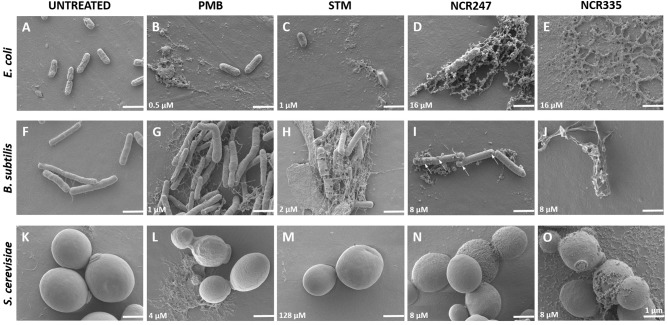
Morphology of control (untreated) and peptide/antibiotics treated *E. coli*, *B. subtilis* and *S. cerevisiae* at MICs observed with Scanning Electron Microscopy. All treatments were made in LSM at minimal inhibitory concentration (MIC) shown in the lower left corner of each panel. The arrows (panel **I**) indicate the observed blebs and blisters. Scale bar: 1 micron.

**FIGURE 2:**
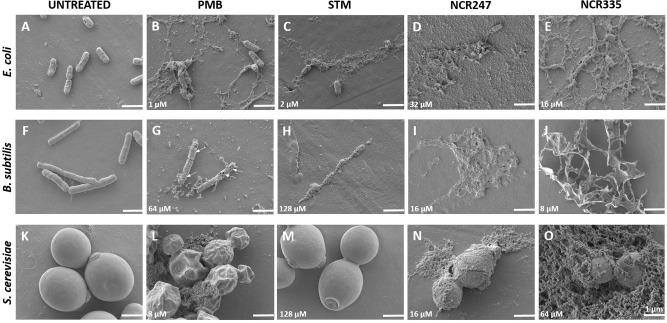
Morphology of control (untreated) and peptide/antibiotics treated *E. coli*, *B. subtilis* and *S. cerevisiae* at MBCs observed with Scanning Electron Microscopy. All treatments were made in LSM at minimal bactericidal concentration (MBC) shown in the lower left corner of each panel. The arrows (panel **G**) point to the holes on the Bacillus cells. Scale bar: 1 micron.

The morphological changes on the cells treated with NCR247, NCR335, PMB and STM in LSM media at MICs and MBCs after 20 h incubation were observed with scanning electron microscopy (SEM) (Figures 1, 2). Compared to the control untreated *E. coli*, *B. subtilis* and *S. cerevisiae* cells (Figures 1A,F,K, 2A,F,K), the various peptide/aminoglycoside treatments provoked drastic morphological alterations in line with their antimicrobial effects. Treatment of *E. coli* cells with PMB and STM at MIC provoked cell lysis although normal cells have also been detected (Figures 1B,C). Clear changes were observed when 16 μM (MIC) NCR247 treatment was applied, bleb formations were observed on the surface of *E. coli* cells (Figure 1D). NCR335 treatment resulted in drastic cell lysis at 16 μM MIC and MBC (Figure 1E). PMB and STM at MIC provoked leakage of *B. subtilis* cells (Figures 1G,H), while NCR247 treatment at 8 μM (MIC) resulted in blisters on the membrane surface (Figure 1I). Also, some cell debris accumulated similarly to that detected in *E. coli* treated with NCR247. Complete cell lysis was observed on *B. subtilis* in response to NCR335 treatment at 8 μM (MIC and MBC) (Figure 1J). PMB at MIC provoked changes in the shape and size of the *S. cerevisiae* yeast cells compared to the untreated samples. The PMB treated cells are more relaxed implying to changes in the membrane structure. The appearance of cell debris around the whole cells also indicates membrane damage likely resulting in altered osmotic pressure (Figure 1L). STM had no effect on yeast, the cells remained intact and showed normal surface even after 20 h incubation (Figure 1M). NCR247 at MIC provoked wrinkly yeast surface and the cells showed the initial signs of lysis (Figure 1N). NCR335 treatment at 8 μM MIC resulted in yeast cell wall degradation (Figure 1O).

All PMB, STM, NCR247 and NCR335 provoked complete cell lysis on *E. coli* at MBC (Figures 2B–E). Large amount of cell debris was observed around the lysed *E. coli* cells. Holes were identified on the cell surface of *B. subtilis* when treated with PMB at MBC (Figure 2G). STM and both NCR peptides completely lysed the *B. subtilis* cells at MBC as observed at 20 h after treatment (Figures 2H,I,J). The loss of turgor pressure and cell lysis were observed on *S. cerevisiae* in response to PMB treatment at 8 μM MBC (Figure 2L). As expected, STM had no visible effect on yeast (Figure 2M), while NCR247 and NCR335 provoked cell lysis at MBC (Figures 2N,O).

## Discussion

The majority of the available data on the activity (either bactericidal or bacteriostatic) of antimicrobial agents is based on endpoint growth determination. Disk diffusion and broth microdilution tests represent the most common standard approaches for testing antibiotic susceptibility of microbes (Jorgensen and Ferraro, 2009). These approaches are almost exclusively used for bacterial sensitivity tests against traditional antibiotics. However, the approaches for testing microbial sensitivity against peptide antibiotics are more diverse. A number of customized approaches have been developed and used for the determination of the antimicrobial effect and spectrum of various peptide molecules (Herigstad et al., 2001; Chen et al., 2003; Theophel et al., 2014). The drop plate technique has been routinely used for activity characterization of peptides (Chen et al., 2003). The main reason for the application of such non-standard techniques is to avoid the generally occurring inactivation of peptide agents by divalent cations present in the growth media. Most cationic peptides simply cannot exert their destructive effects on the electronegative bacterial membranes in normal growth media, as especially Mg^2+^ and Ca^2+^ by interacting with negatively charged lipid moieties inhibit their accessibility to the bacterial membranes. The application of the drop plate technique can bypass this issue, as the living bacteria are harvested in a buffer containing low cation concentration prior to peptide treatment. The treated microorganisms are then dropped on normal growth medium. Thus, the drop plate technique provides data on the survival rate of microbes in a controlled and defined low cation environment. However, this is definitely a compromise and indicates the limitations of the exploitability of most cationic peptide drugs.

The major aim of this study was to compare and visualize the activity and membrane effects of two natural plant peptides (NCR247 and NCR335) in various antimicrobial susceptibility approaches. Two widely used techniques (standard broth microdilution and customized drop plate approach) and three common media (MHB, MHBII and a low-salt medium (LSM)) were employed to compare the antimicrobial effects. Activities of NCR247 and NCR335 as well as streptomycin (STM) and polymyxin B (PMB) were compared against representative microbial species, the Gram-negative *E. coli*, the Gram-positive *B. subtilis* and the budding yeast *S. cerevisiae*.

The standard broth microdilution assay is based on the use of non-cation-adjusted MHB growth medium and the MHBII medium with lowered concentrations of cations. Beside MHB and MHBII, LSM was used to decrease the inhibition effect caused by divalent cations. PMB proved to be the most active against the tested microorganisms, this peptide antibiotics efficiently inhibited and killed *E. coli* and *S. cerevisiae* in all applied media. *B. subtilis* was also strongly inhibited by PMB, however, the MBC value of PMB against Bacillus was quite high (64 μM) in MHB and in LSM as well. As expected, STM was unable to inhibit *S. cerevisiae* in any media, and the NCR peptides showed lower antimicrobial activity against all tested microbes in MHB and MHBII media compared to that obtained in LSM.

The microtiter plate assay is mostly used for endpoint growth determination in susceptibility testing, but growth curve analysis is not in routine use. The investigation of the microbial growth dynamics provides more detailed and specific information about the effects of the tested antimicrobial agents (e. g., growth rate, maximum OD, possible delay of propagation). In our experiments most of the MIC values determined by the standard assays were confirmed by the growth curve measurements. Slightly different MICs were detected only in a few cases. The MICs of STM and PMB for *B. subtilis* in MHB showed minor changes compared to the data detected by the standard protocol. The reason for these alterations is simply the different time-length of the two approaches. The standard protocol was conducted for 20 h, while growth kinetics were tracked for 24 h. More interestingly, the growth curve analysis approach pointed out that the NCR247 and NCR335 plant peptides often exerted a concentration-dependent growth start delay at sub-MICs on the tested microorganisms.

As an alternative testing approach we have used the drop plate method to determine complete elimination (CE) values (Farkas et al., 2017) in phosphate buffer free of divalent cations (2 h incubation). Again, *S. cerevisiae* proved to be fully tolerant to STM. Interestingly, STM was able to eliminate *B. subtilis* only at 128 μM concentration, while NCR247 and NCR335 exerted strong antimicrobial effect against this Gram + representative (16 μM and 2 μM, respectively). PMB was highly effective against *E. coli* and *S. cerevisiae*, complete elimination was achieved at a concentration of 0.5 μM both for Gram- model bacterium and yeast. The low efficiency of STM and PMB against *B. subtilis* indicates that the membrane of this Gram + bacterium is more accessible for the plant NCR peptides. On all tested microbes a more pronounced complete elimination effect was observed for NCR335 (≤ 2 μM) than for NCR247 (16–32 μM), similarly to their MIC and MBC values.

Scanning electron microscopy was applied to visualize the effects of the tested antimicrobials. This approach is very powerful to study antimicrobial agents since SEM is suitable to provide deep visual insight into the membrane disruption and microbial cell killing effects of AMPs. In response to NCR247 treatment at MIC *E. coli* and *B. subtilis* outer membranes were destabilized instantly and peptide penetration resulted in blisters and bleb-like structures as observed in the SEM micrographs. Complete disruption of the inner membrane and the release of the cell content were observed at MBC concentration. MIC value of NCR335 was equal to its MBC value in LSM broth for *E. coli* and *B. subtilis* as well. SEM micrographs confirmed MIC and MBC data, complete bacterial cell lysis was observable at MBC. *S. cerevisiae* was tolerant to STM, no morphological changes could be observed in response to antibiotics treatment. In response to PMB treatment at MIC the original spherical shape of *S. cerevisiae* has been modified. The observed wrinkled surface and the deformation of the cell shape suggested membrane permeabilization and the release of yeast cell plasma when PMB was applied at MBC. NCR peptides induced intense cell degradation and lysis which was confirmed by the appearance of intracellular material around the yeast cells.

**FIGURE 3:**
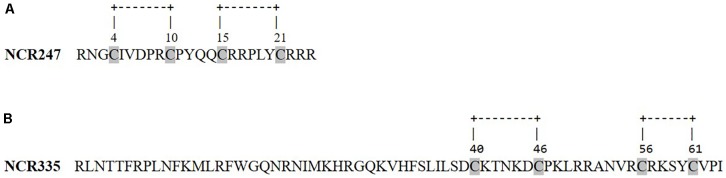
Primary sequences and disulfide bonds of NCR247 **(A)** and NCR335 **(B)** peptides.

It is suggested that the symbiotic NCR peptides might have evolved from the defensins of *M. truncatula* (Maróti et al., 2015). The thorough sequence analysis of the investigated NCR peptides also revealed their clear antimicrobial properties and indeed showed functional and sequence similarity to plant defensins. In plants, the majority of AMPs are abundant in cysteine residues, a feature that enables the formation of two to six disulfide bonds (Tam et al., 2015). The role of cysteine residues in the NCR peptides is particularly important similarly to that in defensins. Two or three disulfide bonds contribute to the structure of NCRs and to their resistance against proteolytic, thermal and chemical degradation (Aoki and Ueda, 2013). NCR247 and NCR335 both possess two disulfide bridges (Figures 3A,B) (Farkas et al., 2017). It was shown for NCR247 that either the replacement of the cysteine residues or the modification of the disulfide bonds altered the peptide’s antimicrobial activity (Haag et al., 2012). NCR247 and NCR335 peptides are also abundant in cationic amino acid residues, especially in arginine. The high positive net charge is associated with the ability to induce membrane depolarization and disintegration of both Gram-negative and Gram-positive bacteria (Arikan, 2007; Farkas et al., 2017). Based on thorough sequence analyses and various antimicrobial tests NCR335 proved to be a generally more potent antimicrobial agent compared to NCR247, which can be at least partly explained by the higher net charge of NCR335 (+ 14 vs. + 6) (Farkas et al., 2017). Also, the more hydrophobic character of NCR335 (29 and 37% hydrophobic amino acids for NCR247 and NCR335, respectively) may contribute to its higher antimicrobial efficiency.

The basic conclusion of our work is that assay parameters - especially the applied medium in which the microbes are treated - strongly influence the antimicrobial efficiency of peptide agents. This sensitivity of the cationic peptides for the presence of divalent cations in the applied medium is a general observation; however, the extent of dependence is different for the various peptides (activity of PMB is less dependent, while NCR247 is strongly dependent). This unique feature of the peptides has to be considered when future medical applications of selected peptides are planned. Our specific results showed that the natural plant peptide NCR335 can be considered as a strong antimicrobial agent having a general efficiency against bacteria and yeast comparable to that of PMB (polymyxin B). NCR335 even outperformed PMB under certain conditions; lower MBC value of this plant peptide was effective against *B. subtilis* either in MHBII and LSM. Similarly, complete elimination of *B. subtilis* was achieved by 2 μM NCR335 using the drop plate method, while high tolerance to PMB was observed in this bacterium under the same conditions.

## Author Contributions

GM and AF designed and executed the experiments as well as composed the manuscript. BP carried out the experiments. ÉK added useful recommendations and edited the manuscript.

## Conflict of Interest Statement

The authors declare that the research was conducted in the absence of any commercial or financial relationships that could be construed as a potential conflict of interest.
