# Supplementary material for: Antimicrobial Activity of NCR Plant Peptides Strongly Depends on the Test Assays
Source: Front Microbiol. 2018 Oct 30;9:2600. doi: 10.3389/fmicb.2018.02600 (PMC6218624; doi:10.3389/fmicb.2018.02600)
Supplement: Supplementary file 1 [file Data_Sheet_1.PDF]

Supplementary Material

Antimicrobial activity of NCR plant peptides strongly depends on the test assays

Attila Farkas, Bernadett Pap, Éva Kondorosi, Gergely Maróti*

Institute of Plant Biology, Biological Research Center of the Hungarian Academy of Sciences, Szeged, Hungary

*** Correspondence: Gergely Maróti**

Institute of Plant Biology, Biological Research Center of the Hungarian Academy of Sciences

Temesvári krt. 62.

Szeged, 6726, Hungary

maroti.gergely@brc.mta.hu

Supplementary Figures

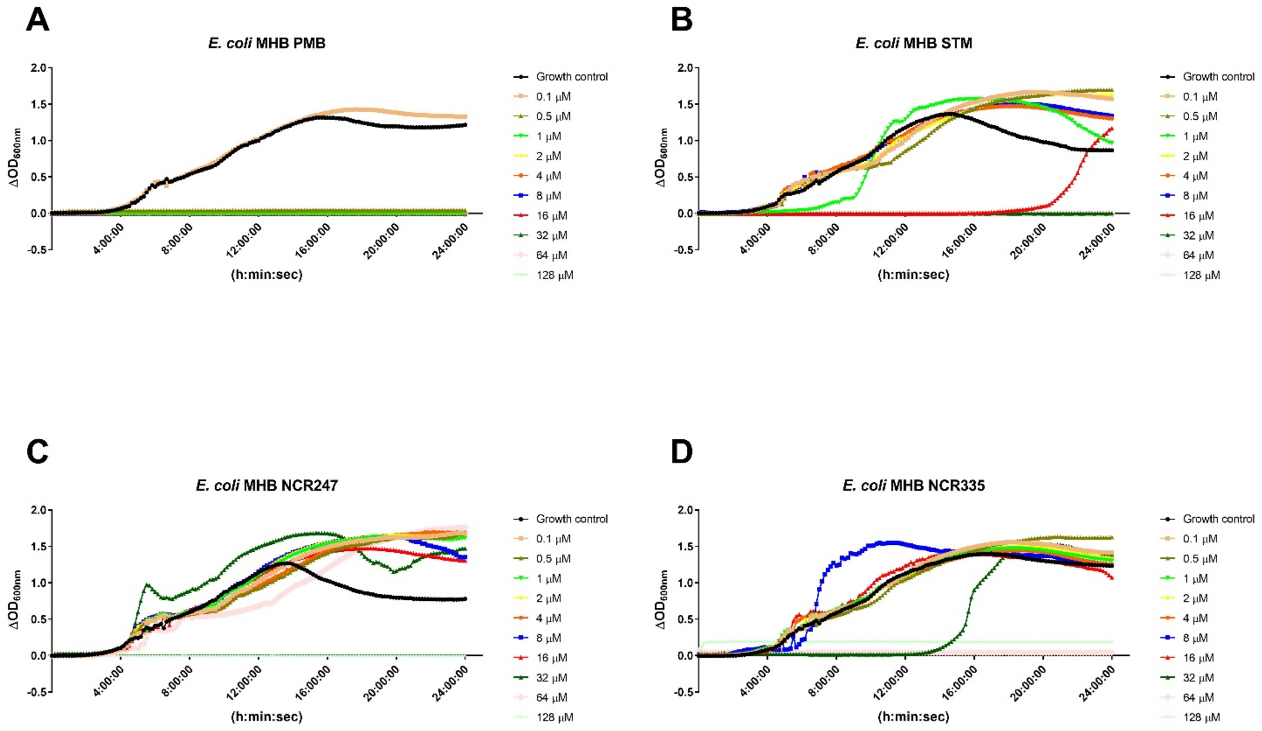

Supplementary Figure 1. Effect of PMB, STM and two NCR peptides on the growth dynamics of *E. coli* in MHB.

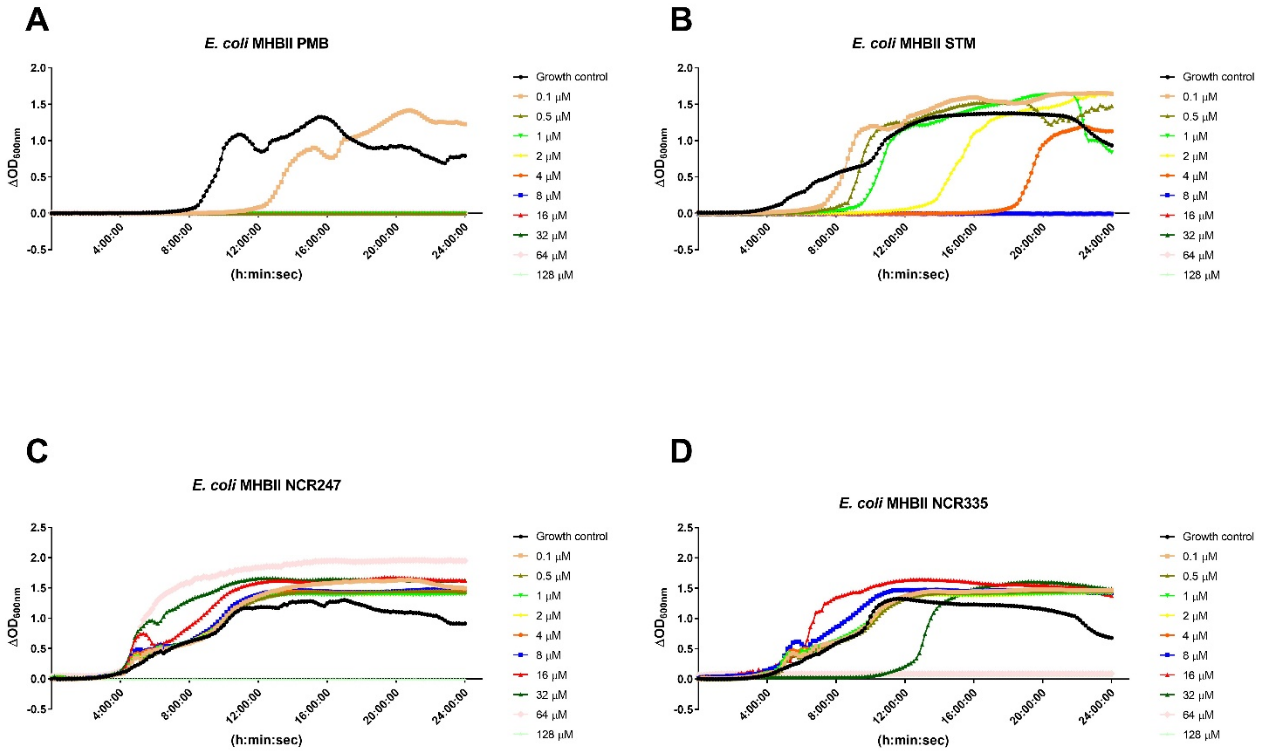

Supplementary Figure 2. Effect of PMB, STM and two NCR peptides on the growth dynamics of *E. coli* in MHBII.

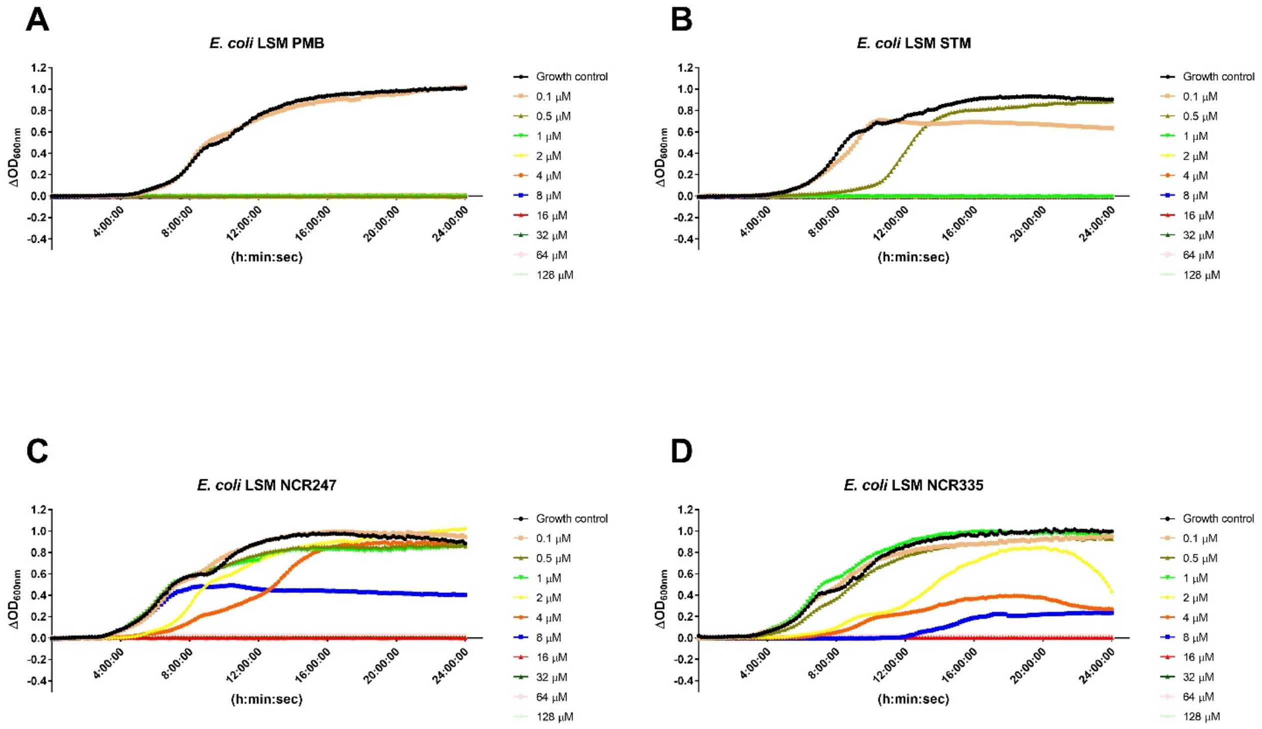

Supplementary Figure 3. Effect of PMB, STM and two NCR peptides on the growth dynamics of *E. coli* in LSM.

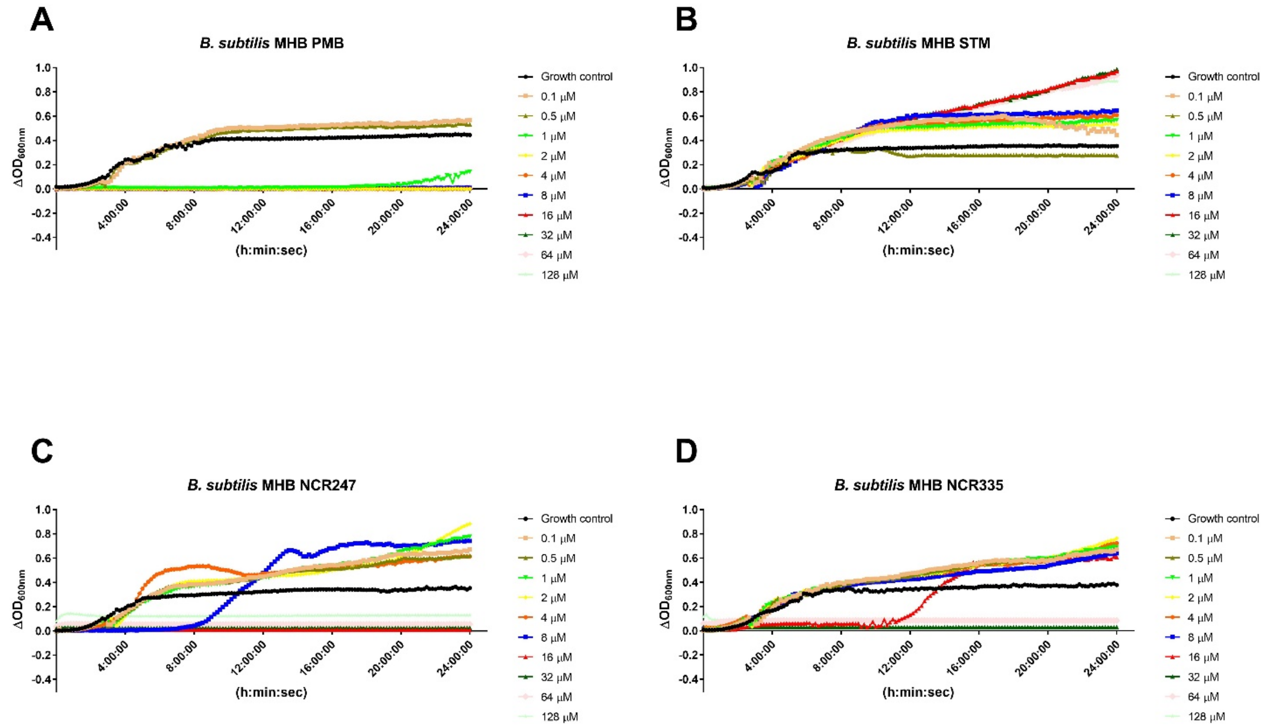

Supplementary Figure 4. Effect of PMB, STM and two NCR peptides on the growth dynamics of *B. subtilis* in MHB.

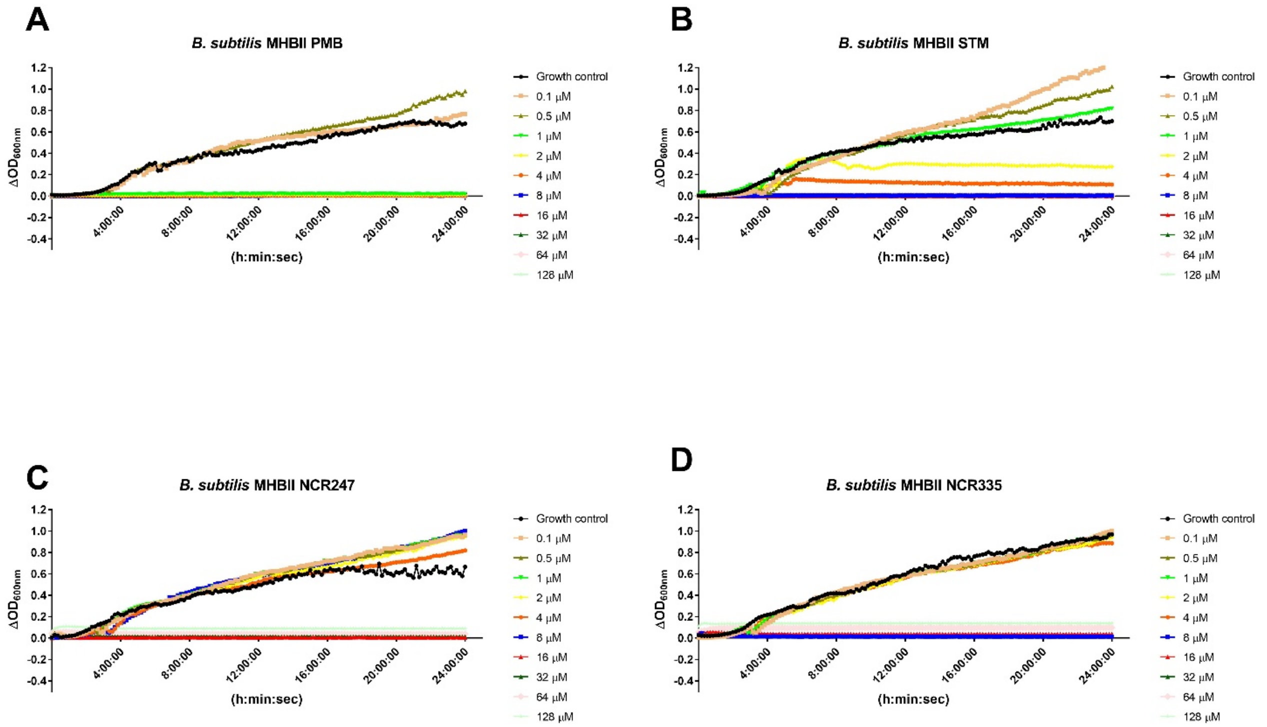

Supplementary Figure 5. Effect of PMB, STM and two NCR peptides on the growth dynamics of *B. subtilis* in MHBII.

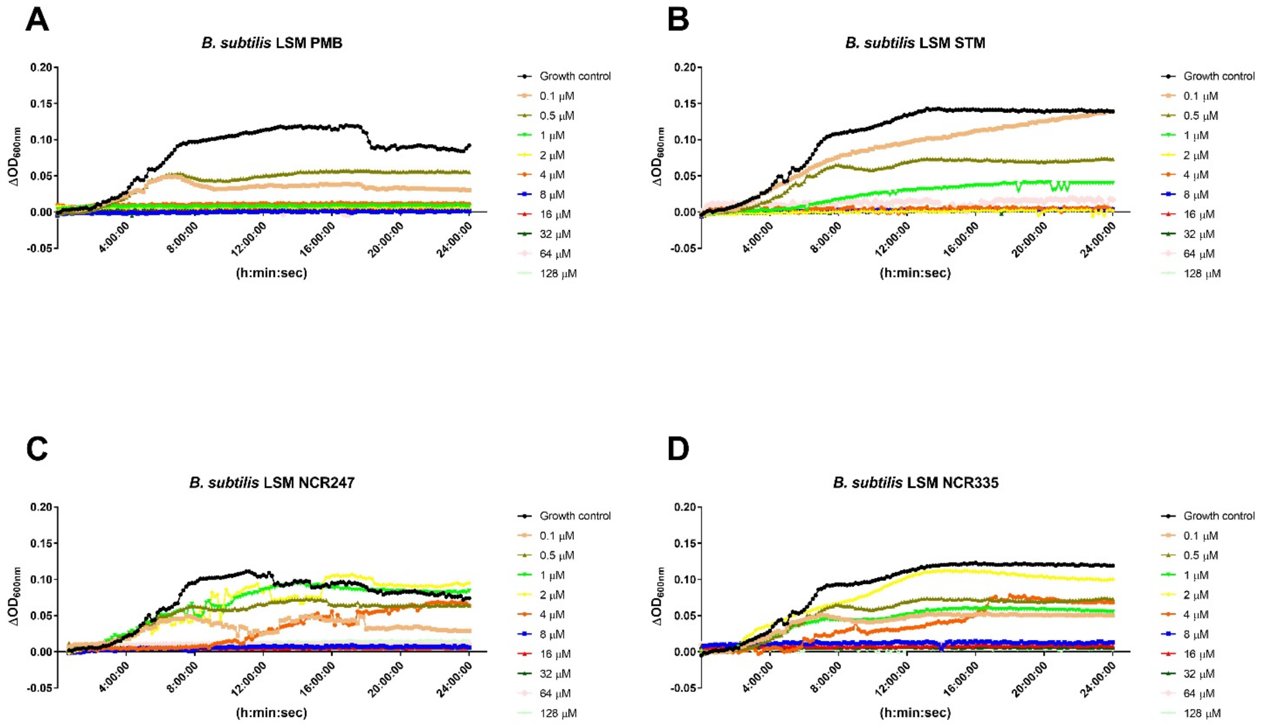

Supplementary Figure 6. Effect of PMB, STM and two NCR peptides on the growth dynamics of *B. subtilis* in LSM.

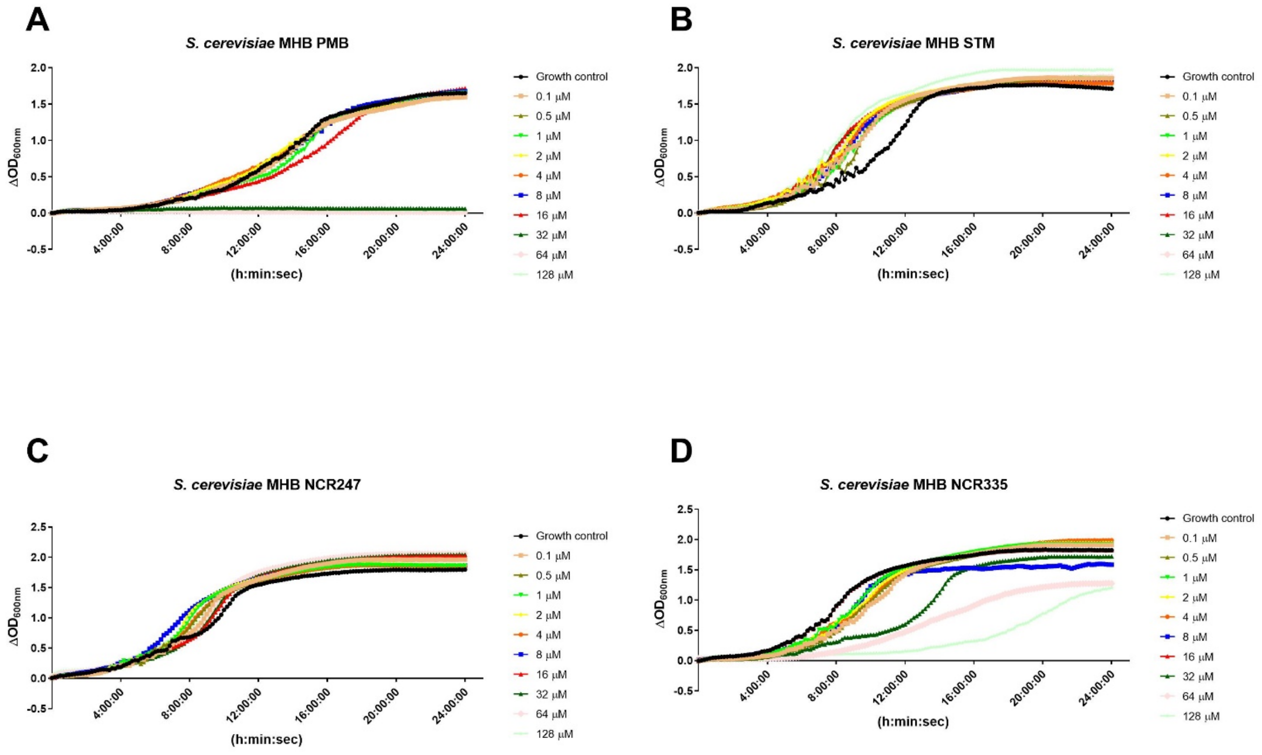

Supplementary Figure 7. Effect of PMB, STM and two NCR peptides on the growth dynamics of *S. cerevisiae* in MHB.

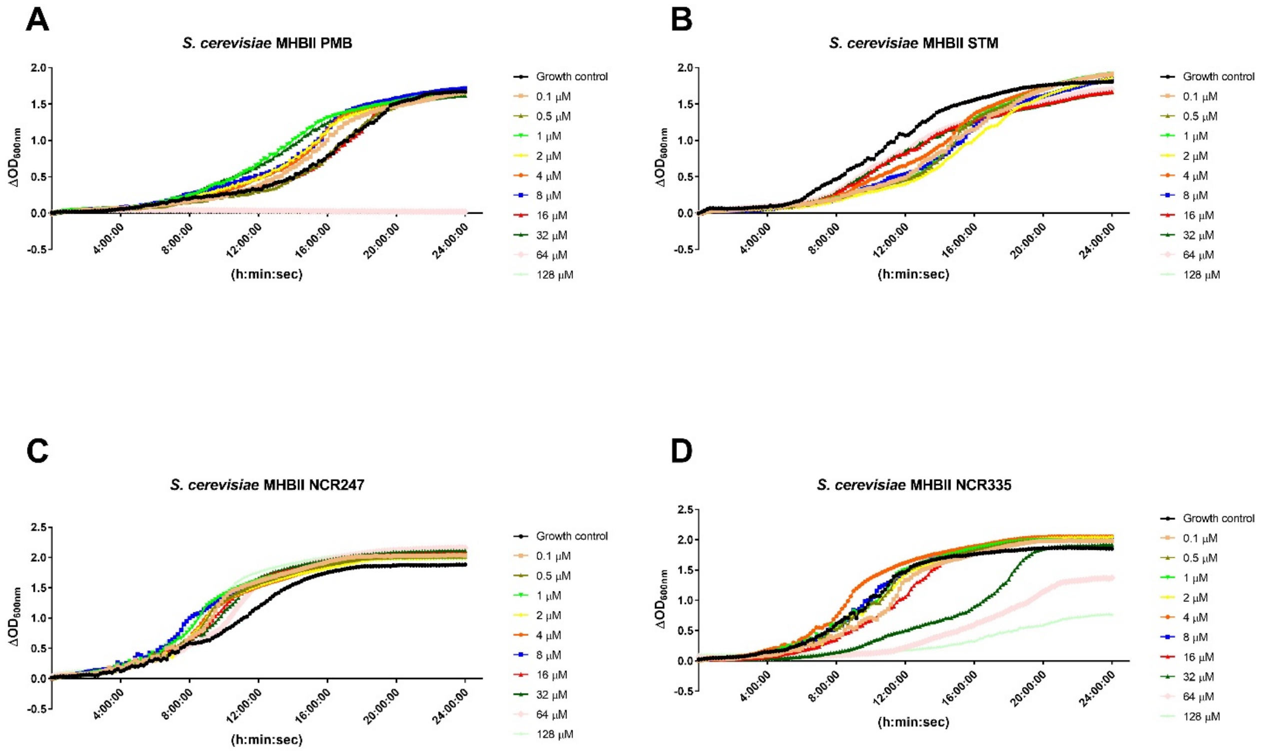

Supplementary Figure 8. Effect of PMB, STM and two NCR peptides on the growth dynamics of *S. cerevisiae* in MHBII.

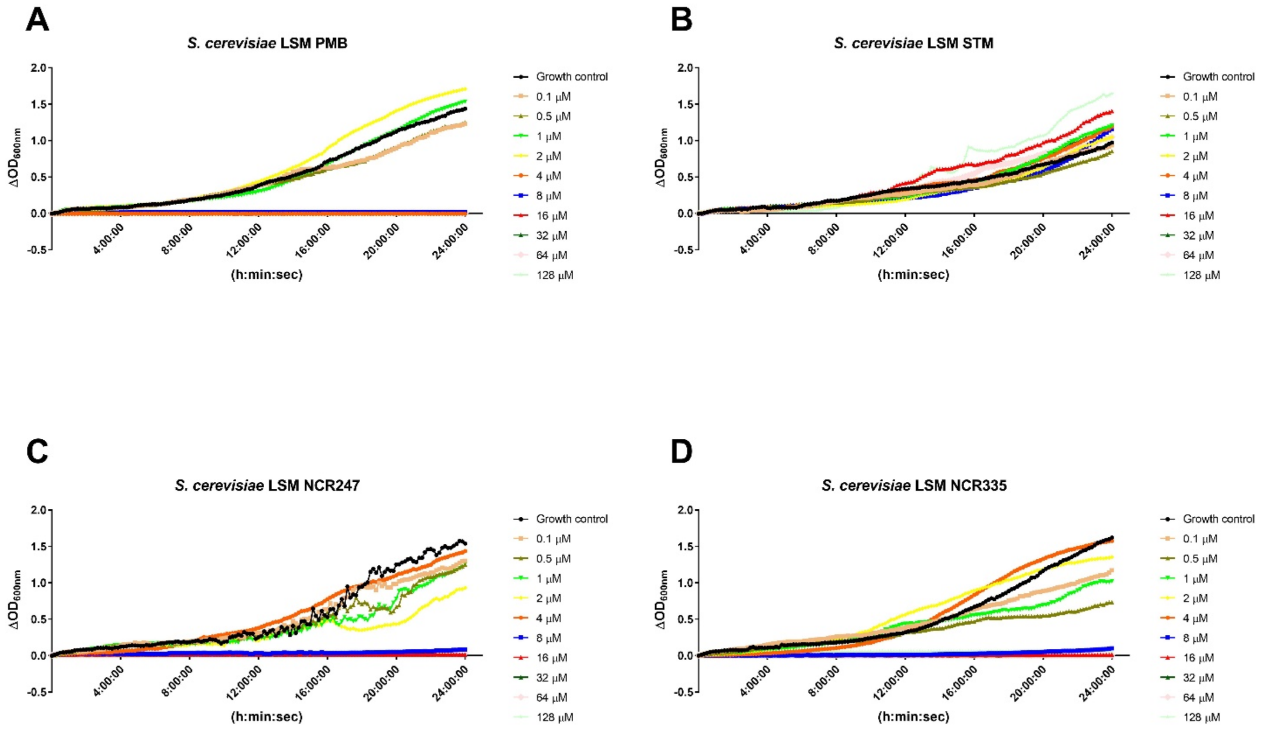

Supplementary Figure 9. Effect of PMB, STM and two NCR peptides on the growth dynamics of *S. cerevisiae* in LSM.
